# Supplementary figures and images for: A subcellular map of the human kinome
Source: eLife. 2021 May 14;10:e64943. doi: 10.7554/eLife.64943 (PMC8175086; doi:10.7554/eLife.64943)

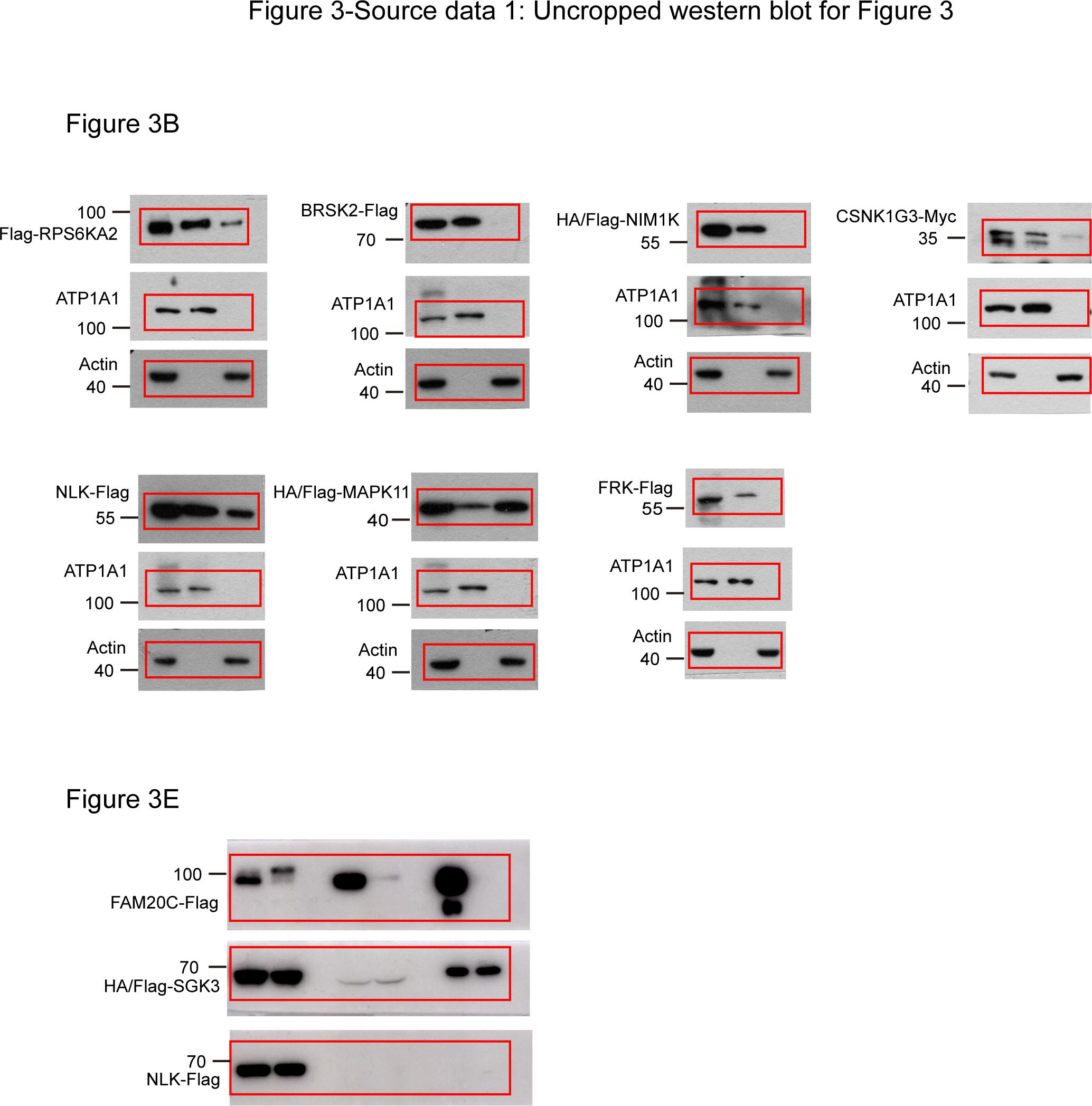

Supplement: Figure 3—source data 1. [file elife-64943-fig3-data1.jpg]

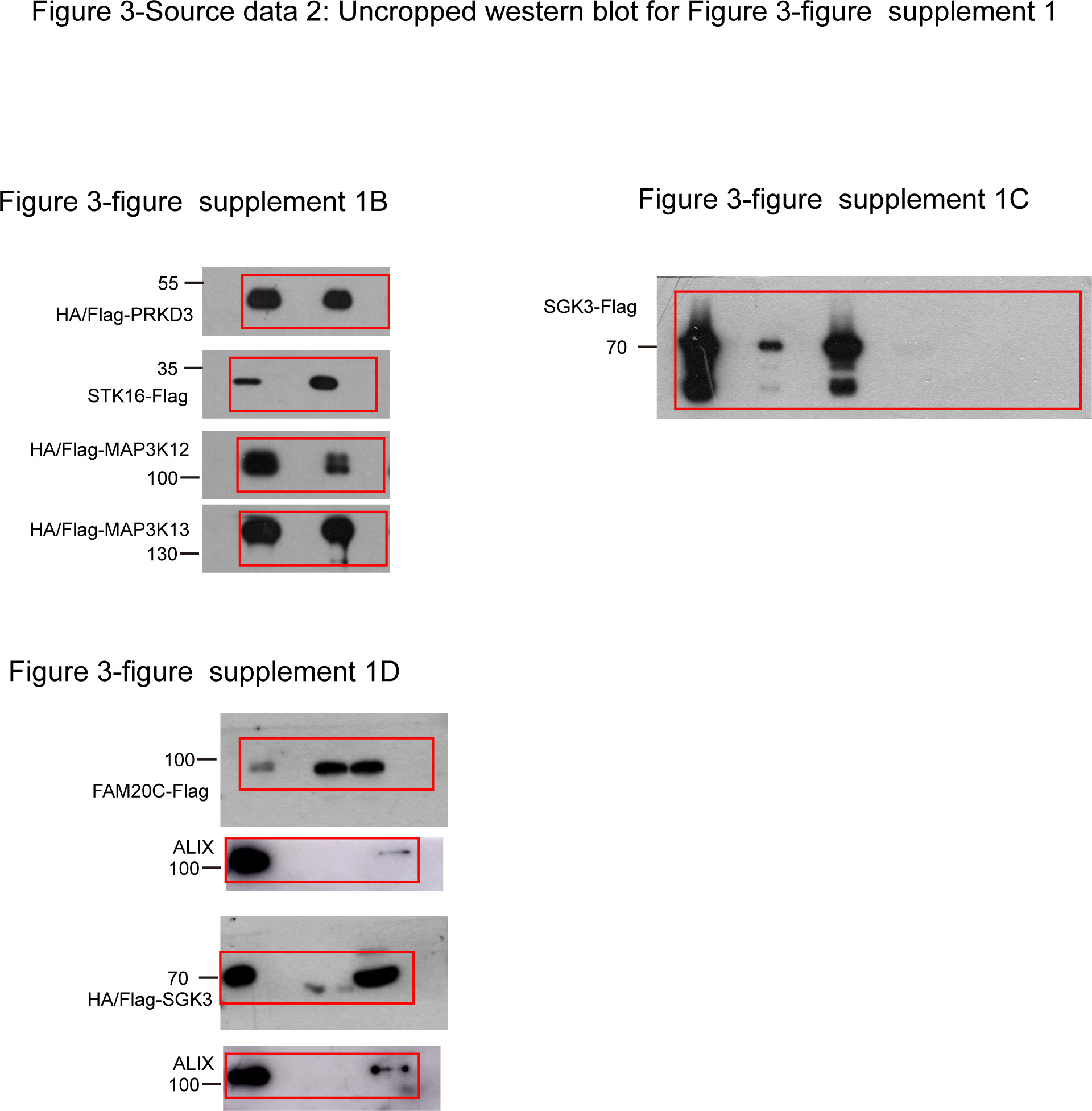

Supplement: Figure 3—figure supplement 1—source data 1. [file elife-64943-fig3-figsupp1-data1.jpg]

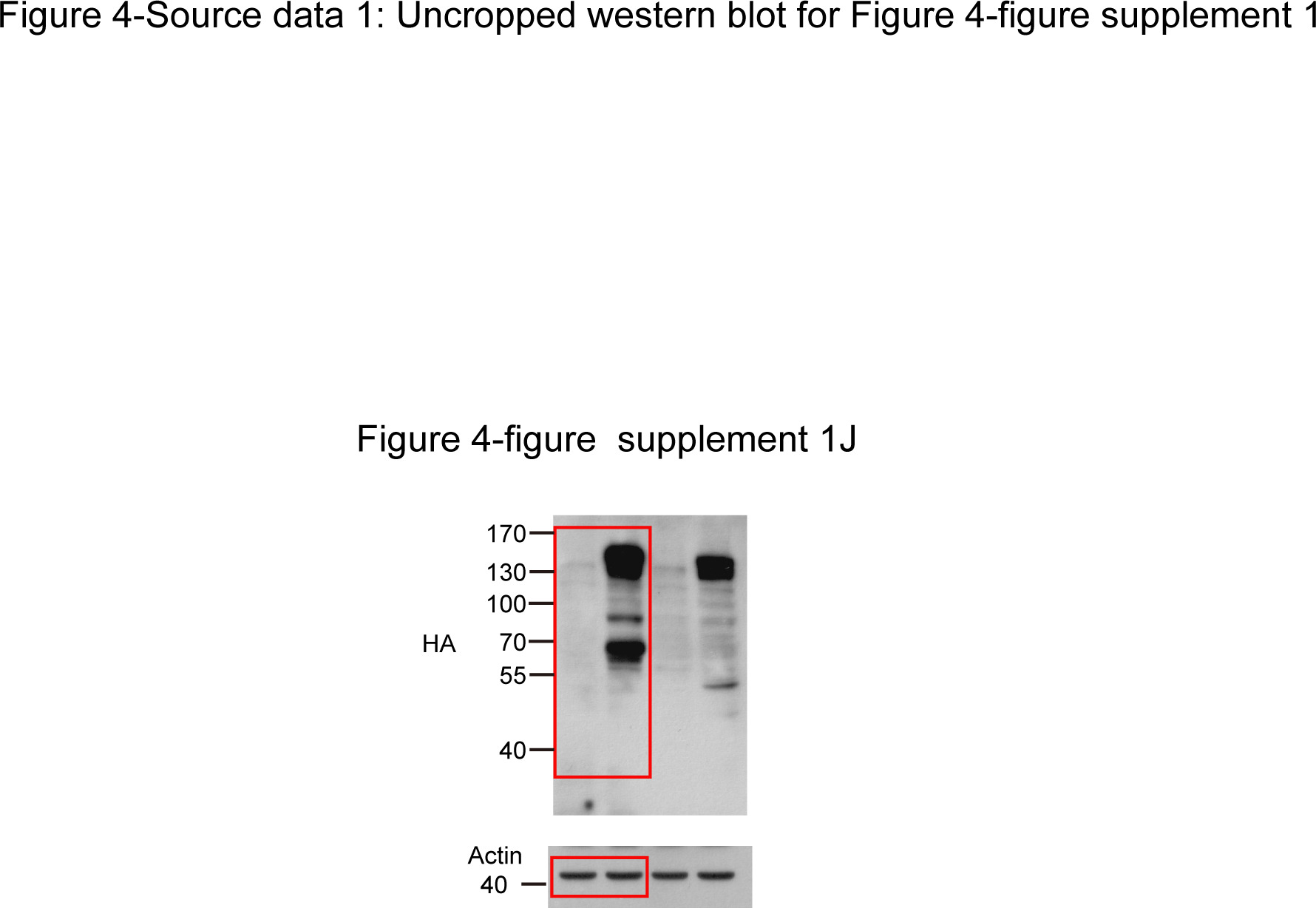

Supplement: Figure 4—figure supplement 1—source data 1. [file elife-64943-fig4-figsupp1-data1.jpg]

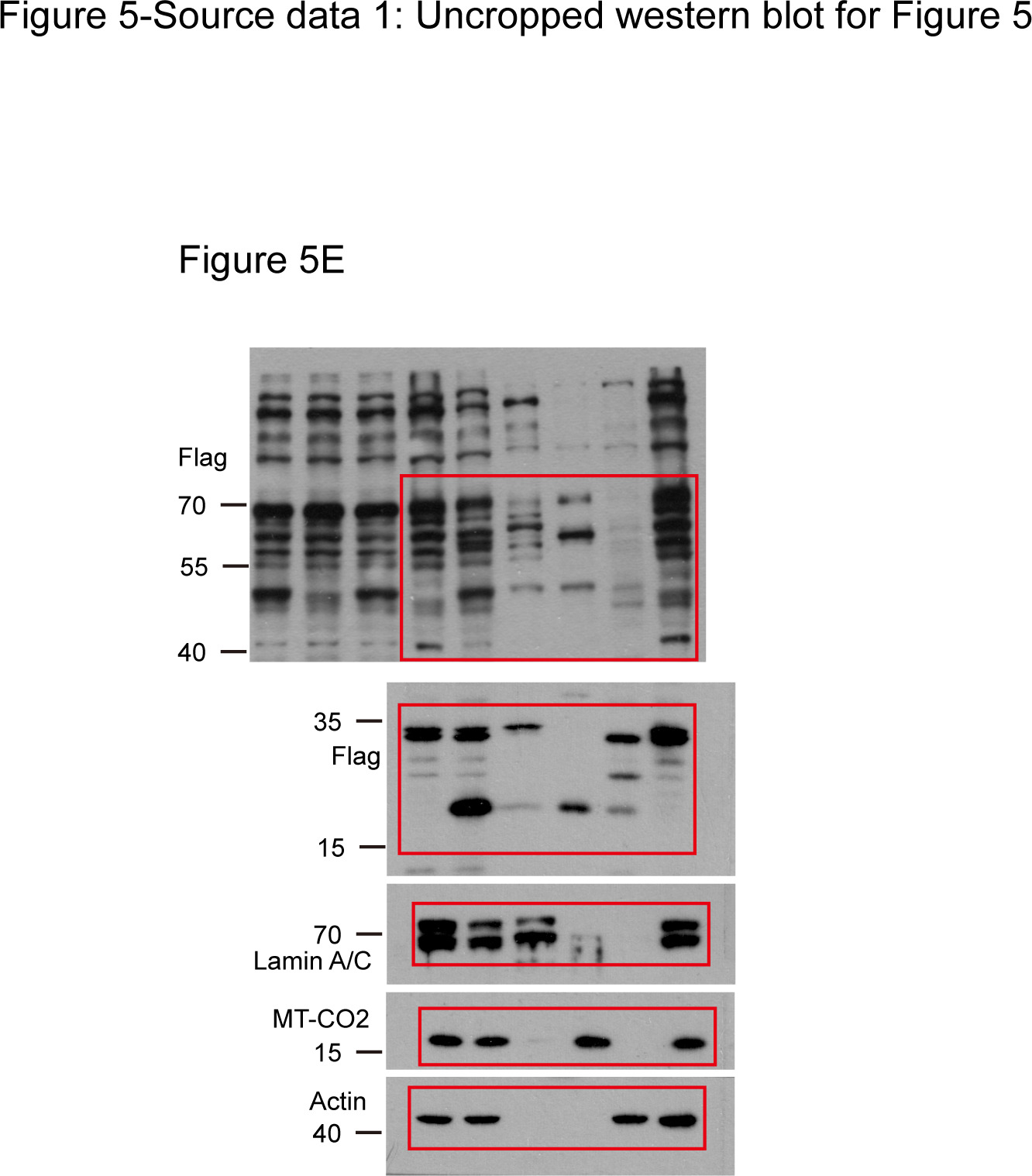

Supplement: Figure 5—source data 1. [file elife-64943-fig5-data1.jpg]

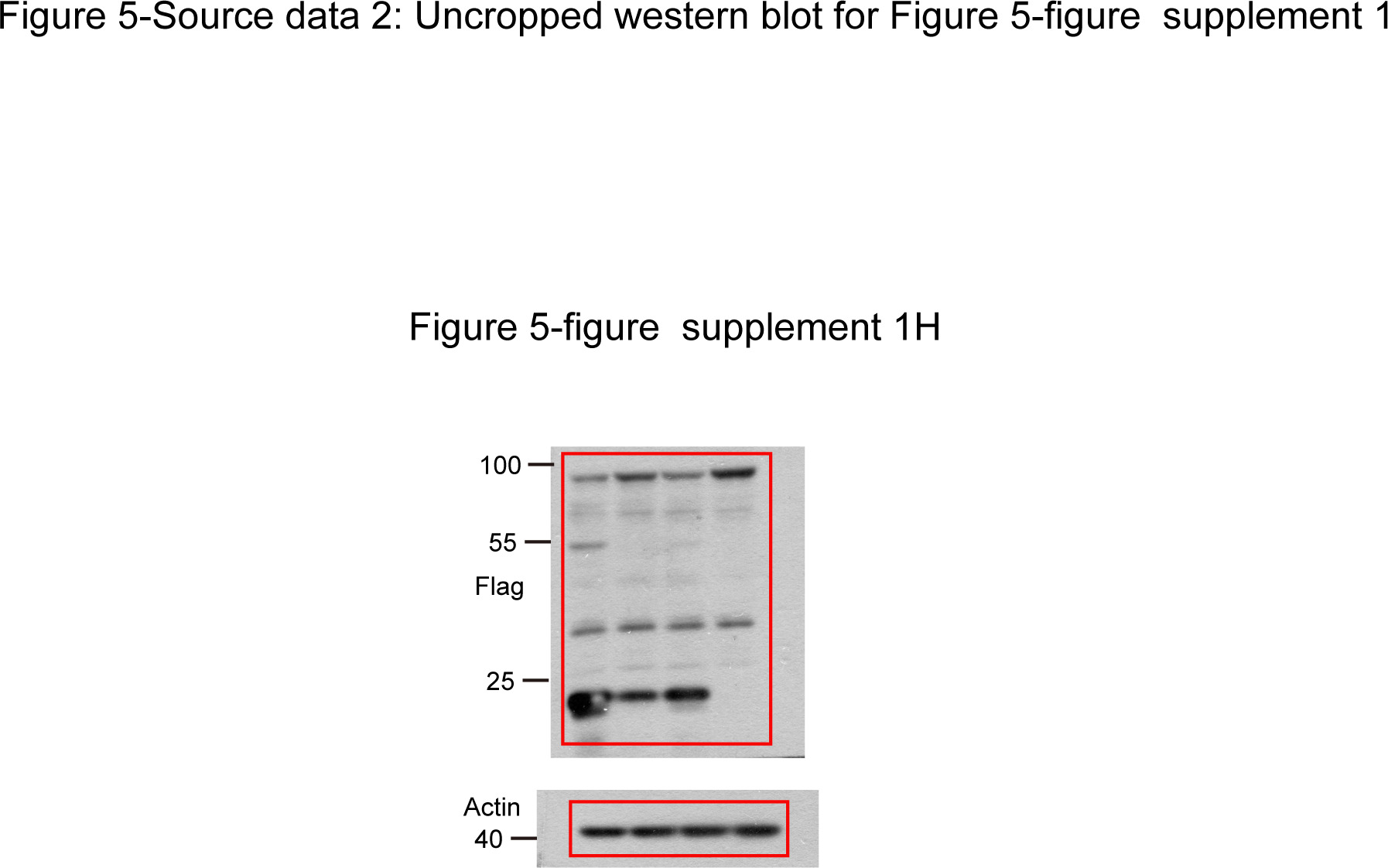

Supplement: Figure 5—figure supplement 1—source data 1. [file elife-64943-fig5-figsupp1-data1.jpg]

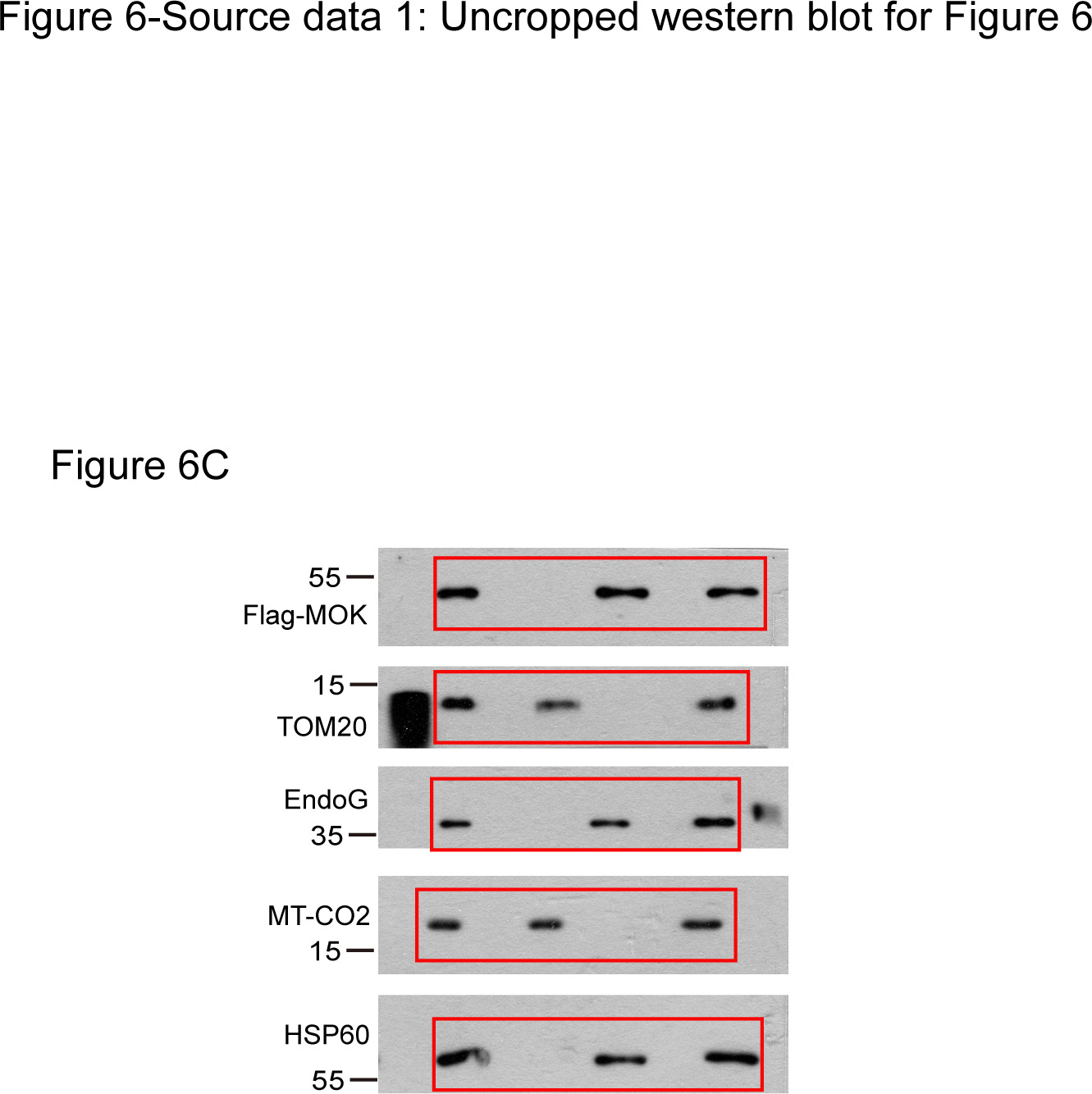

Supplement: Figure 6—source data 1. [file elife-64943-fig6-data1.jpg]

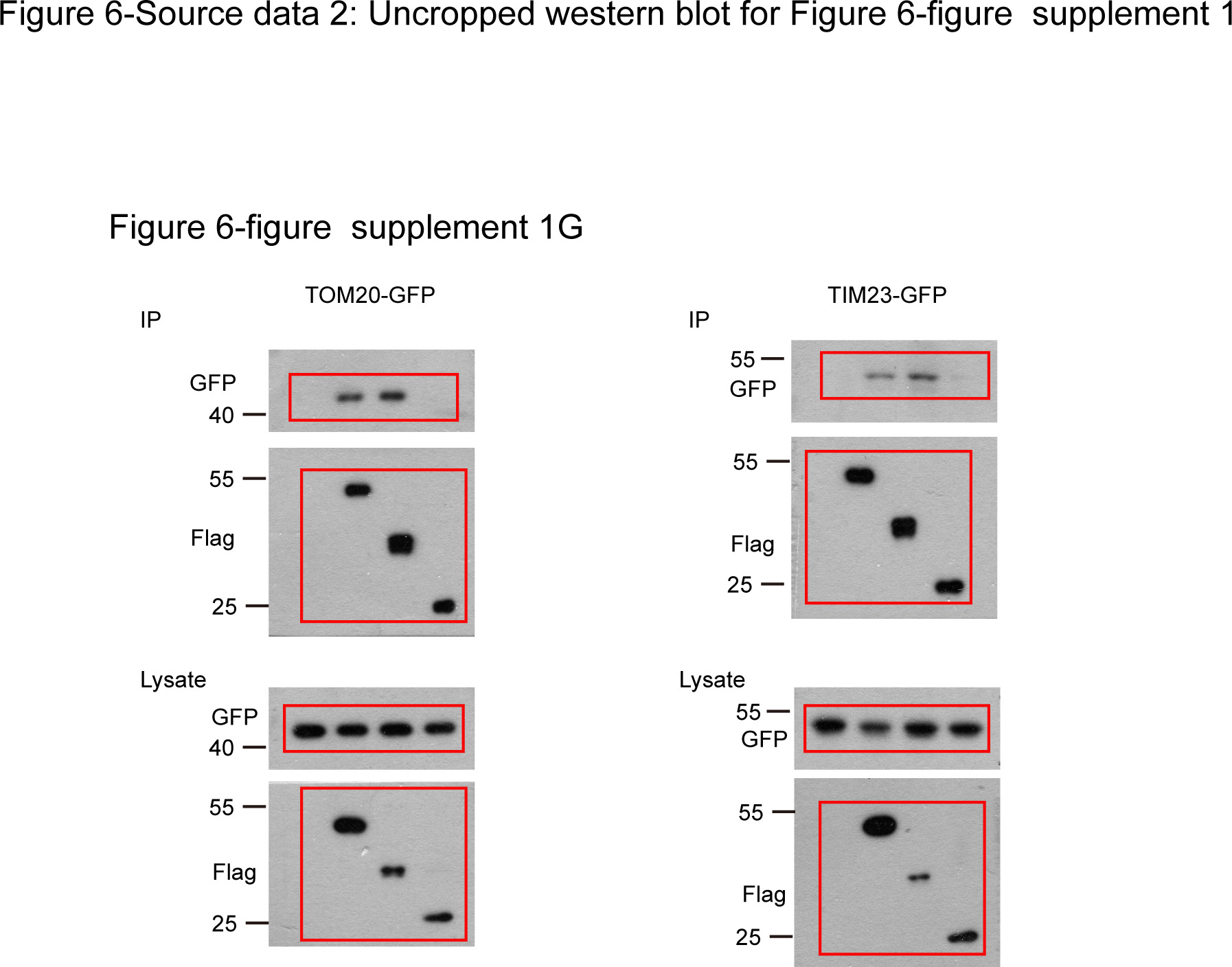

Supplement: Figure 6—figure supplement 1—source data 1. [file elife-64943-fig6-figsupp1-data1.jpg]

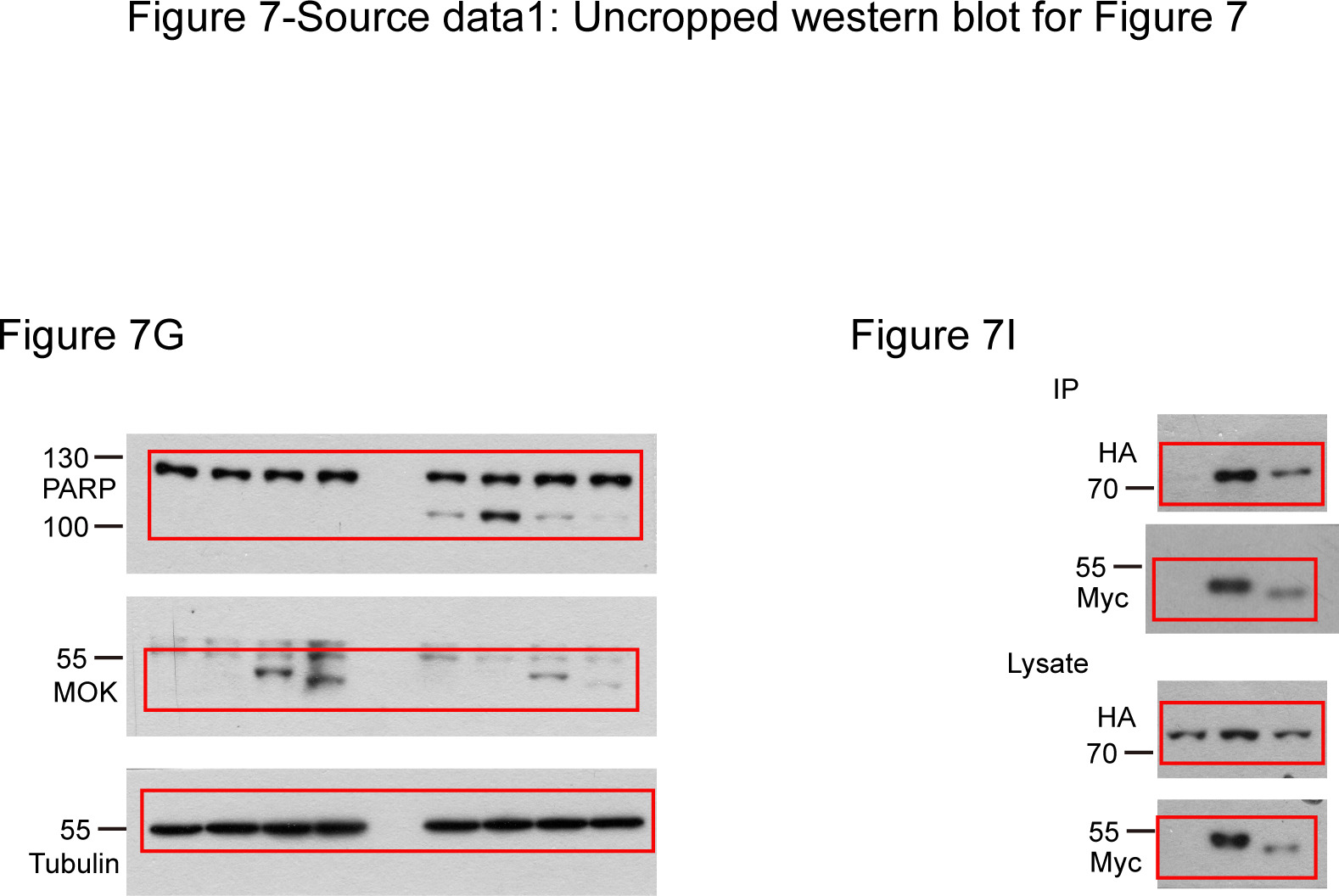

Supplement: Figure 7—source data 1. [file elife-64943-fig7-data1.jpg]

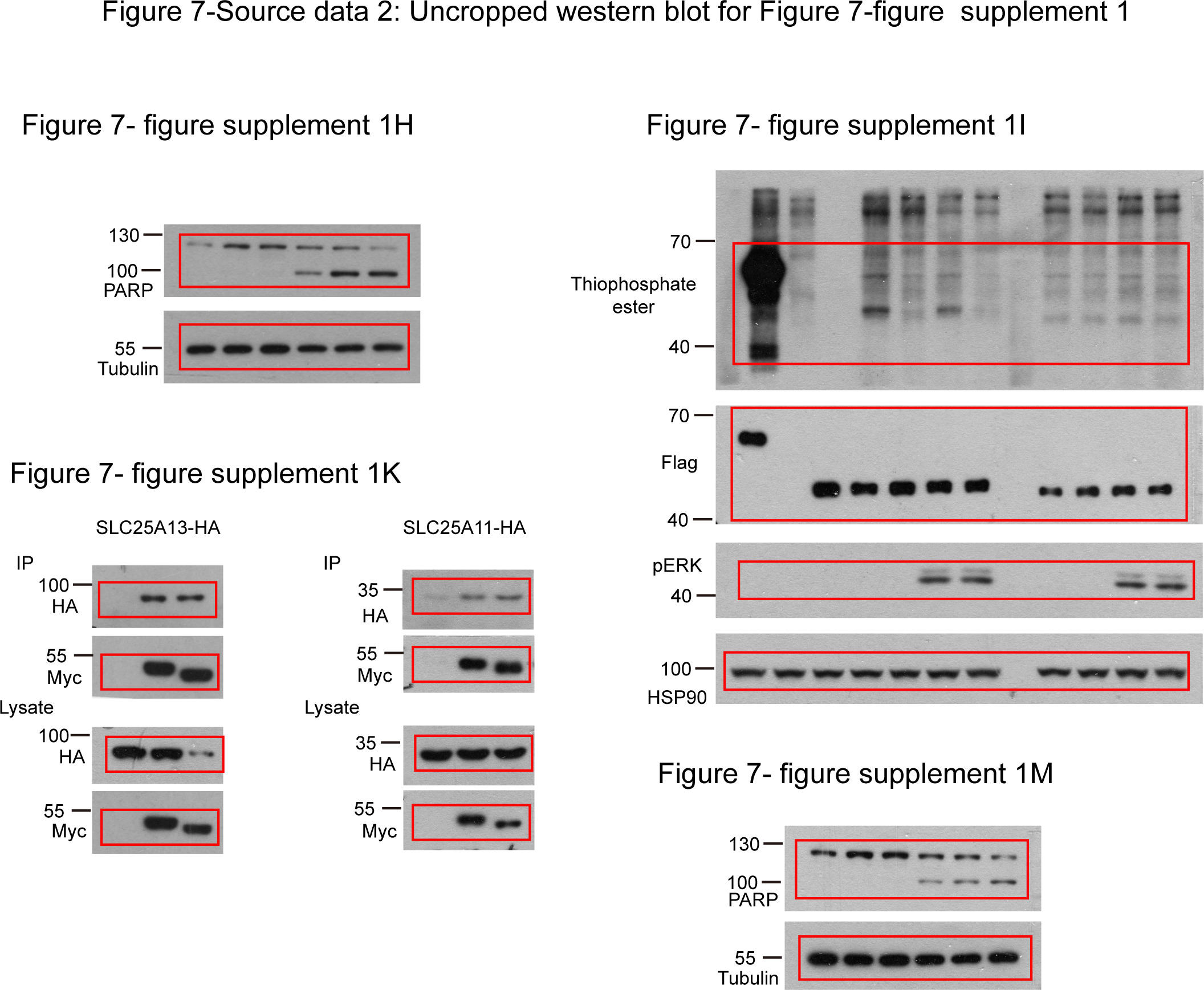

Supplement: Figure 7—figure supplement 1—source data 1. [file elife-64943-fig7-figsupp1-data1.jpg]
